# The Role of Membranes in Tissue Regeneration Therapy: State of the Art and Future Perspectives

**DOI:** 10.1021/acsomega.6c07202

**Published:** 2026-10-06

**Authors:** Raphael Ferreira da Silva, Renata Ribeiro de Castro, Gabriela Warwar Teixeira, Paula de Souza Soares, Valéria Pereira de Sousa, Carlos Rangel Rodrigues, Flávia Almada do Carmo, Lucio Mendes Cabral

**Affiliations:** † Department of Drugs and Pharmaceutics, Faculty of Pharmacy, 314177 Universidade Federal do Rio de Janeiro, Rio de Janeiro 21941-853, Brazil; ‡ Instituto de Tecnologia em Fármacos, Fundação Oswaldo Cruz, Rio de Janeiro 21041-250, Brazil

## Abstract

Membranes for tissue regeneration are an indispensable class of biomaterials in regenerative medicine, serving as selective barriers and matrices for the reconstruction of various tissues. Essential characteristics for the clinical success of a biomaterial include its biocompatibility, degradation kinetics, mechanical properties, and bioactivity, which are the focus of this review, with an emphasis on the importance of immunomodulation and the host response. The main clinical applications of this class of biomaterials are directed towards bone, cartilage, skin, and neural regeneration. In addition to evaluating the essential characteristics of these membranes, innovations such as smart, stimuli-responsive, and bioelectric membranes, as well as advances in 4D bioprinting, were also addressed. It is concluded that, despite established clinical success, the main challenges lie in overcoming material variability, vascularizing large-volume defects, and translating advanced technologies into clinical practice. Future perspectives point towards the development of personalized, smart, and bio-instructive membranes capable of actively orchestrating the regeneration process.

## Graphical abstract

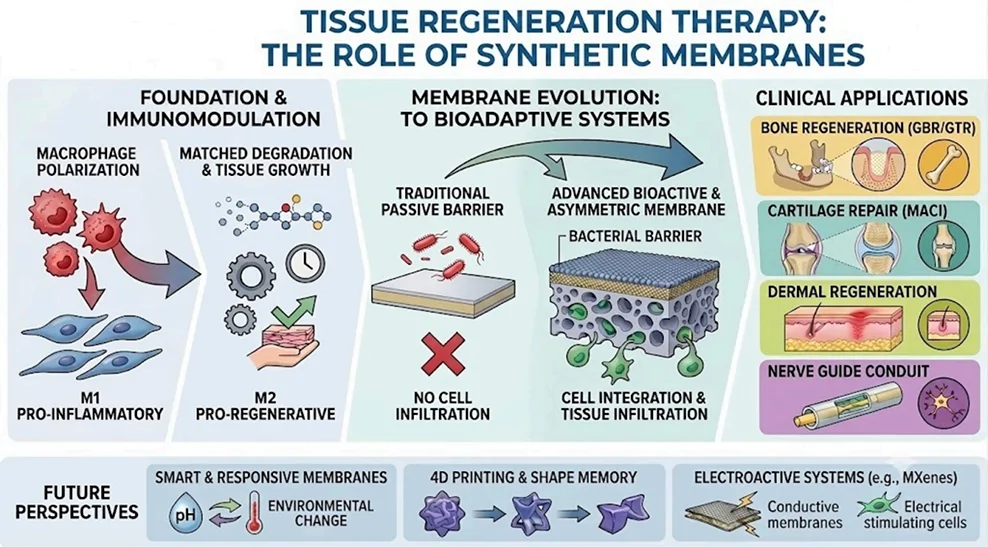

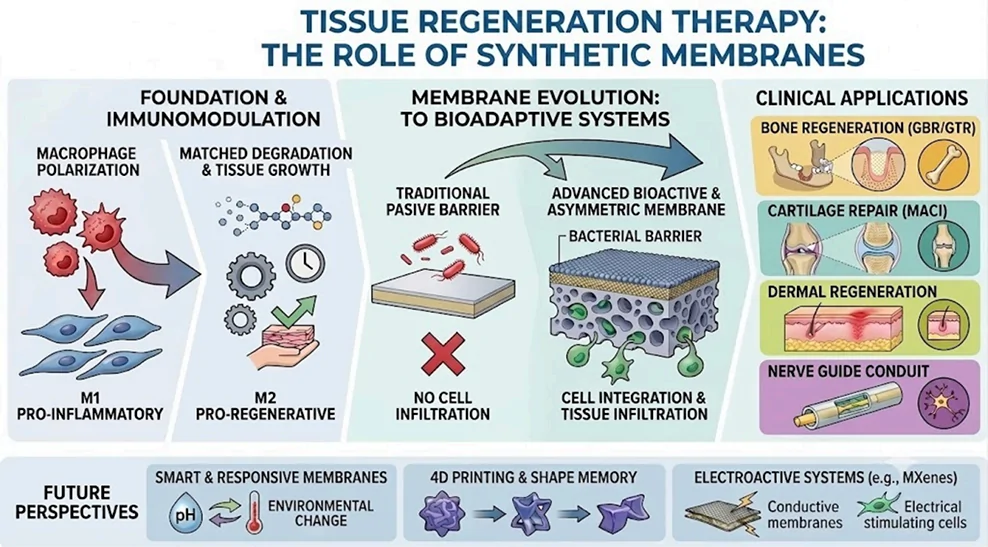

## 1 Introduction

The loss of tissue function, resulting from trauma, chronic diseases such as osteoarthritis and diabetes, congenital anomalies, or the natural aging process, represents a clinical challenge with a major impact on public health.^1^ Traditional therapeutic approaches, although often effective in symptom management, frequently rely on replacing damaged tissue with inert prosthetic materials or tissue transplants, which face barriers such as limited donor availability and morbidity.^2,3^ This reality has driven the search for innovative solutions, including new clinical approaches using tailored responsive biomaterials.^4^ In this context, regenerative medicine has emerged as a field of new technologies focused on the repair, replacement, or regeneration of cells, tissues, and organs to restore their functionality using these new materials.^5^ Unlike conventional treatments, regenerative medicine seeks to harness and amplify the body’s own recovery mechanisms.^6^ This interdisciplinary area integrates knowledge from cellular and molecular biology and materials engineering to develop therapies that can effectively regenerate tissues rather than merely repair them.^7,8^

One of the fundamental pillars of biomaterial use in regenerative medicine is biological tissue engineering, which is based on an interacting triad of components: (i) cells, which are the building blocks of the new tissue; (ii) bioactive signals (e.g., growth factors), which instruct cells on how to behave; and (iii) matrices that provide structural support in an environment conducive to regeneration.^9^ Matrices are particularly crucial, as they act as a temporary substitute for the extracellular matrix (ECM), modulating the three-dimensional (3D) structure of the new tissue and mediating cellular interactions.^10,11^ Among the different types of matrices, membranes stand out as a class of biomaterials with a promising regenerative function. In their most fundamental conception, a membranes work as a selective physical barrier.^12^ By isolating a specific tissue, the membrane prevents the invasion of fast-growing and unwanted cell types, such as epithelial or connective cells, creating a protected compartment where target tissue-specific precursor cells (e.g., osteoblasts) can proliferate and regenerate it in an organized manner.^13,14^

The history of regenerative membranes in periodontics, for example, can be seen as an evolution over time. The first generation consisted of bioinert and nonresorbable materials, such as expanded polytetrafluoroethylene (PTFE) or cellulose, whose sole purpose was to act as a barrier.^15^ The second generation introduced the concept of biodegradability, using polymers such as collagen (marketed under the Bio-Gide brand) and PLGA (poly­(lactic-*co*-glycolic acid)), which are gradually replaced by new tissue, eliminating the need for a second surgery after implantation.^16,17^ Currently, the third generation of membranes stands out, as it is designed to be bioactive, releasing ions or compounds, such as growth factors, that actively stimulate cellular and tissue responses.^18,19^ The growing sophistication of these devices has raised a relevant point, namely, that there is no universal membrane.^20,21^ Failure to match material properties to application needs is one of the main causes of clinical failure.^22,23^

The first and most crucial axis of structural design is biocompatibility, defined by Williams (2008) as “the ability of a material to perform with an appropriate host response in a specific application”. Initially, the goal was bioinertness, that is, to minimize any host response. Today, the paradigm has shifted to active immunomodulation. When a material is implanted, the body invariably recognizes it as foreign and triggers a cascade of events known as the foreign body response , in which macrophages are the central cells. It is understood that the immune response, especially macrophage polarization, is a determining factor in regeneration outcomes.^11,24^ Therefore, the ideal innovative membrane must be able to actively guide the immune response toward a pro-regenerative (M2) phenotype, a concept explored in depth in recent reviews.^14,25^ The review by Gou et al. reinforced the centrality of macrophages in the GBR, highlighting that the most effective membranes are potent immunomodulators rather than passive barriers.^14^ Additionally, Mei et al. showed that the matrix stiffness of a membrane is a crucial mechanical factor that, by itself, can cause macrophage polarization, an emerging field called mechano-immunomodulation.^11^ Nanotechnology intensified this approach by enabling manipulation of surfaces at the submicrometric scale, influencing protein adsorption and immune cell polarization to favor regenerative processes. Thus, materials began to be conceived not only as passive supports but as therapeutic tools capable of inducing specific cellular responses and actively contributing to the functional restoration of tissues.

The second critical axis of structural design involves balancing mechanical properties with the biodegradation rate. The membrane must be mechanically robust enough to maintain the regenerative space yet flexible enough for surgical handling.^26^ Simultaneously, its biodegradation rate must be synchronized with the formation rate of new tissue, a complex challenge that depends on the interaction between the material’s properties and the local physiological environment.^16,27^ The third axis of structural design concerns bioactivity, the membrane’s ability to interact with target tissue cells. This ranges from passive osteoconductivity, where the membrane serves as a scaffold for bone growth, to active osteoinductivity, induced by the release of growth factors.^26^ The most advanced strategies, as highlighted in 2024 reviews, involve dual- or sequential-release systems and “smart” membranes that release therapeutic agents in response to physiological stimuli.^19,28^ Given this complexity and the need to optimize and harmonize these three fundamental axes, the objective of this work was to conduct a narrative and critical review of the state of the art in membranes for applications in tissue regeneration therapy.

## 2 Classification of Tissue Regeneration Membranes

There is a wide variety of materials (Figure 1) and membrane preparation techniques for tissue regeneration.^29^ Membranes can be classified according to multiple criteria, including the origin of their constituent materials, their morphological structure, and the method by which they are manufactured.^20^

**Figure 1:**
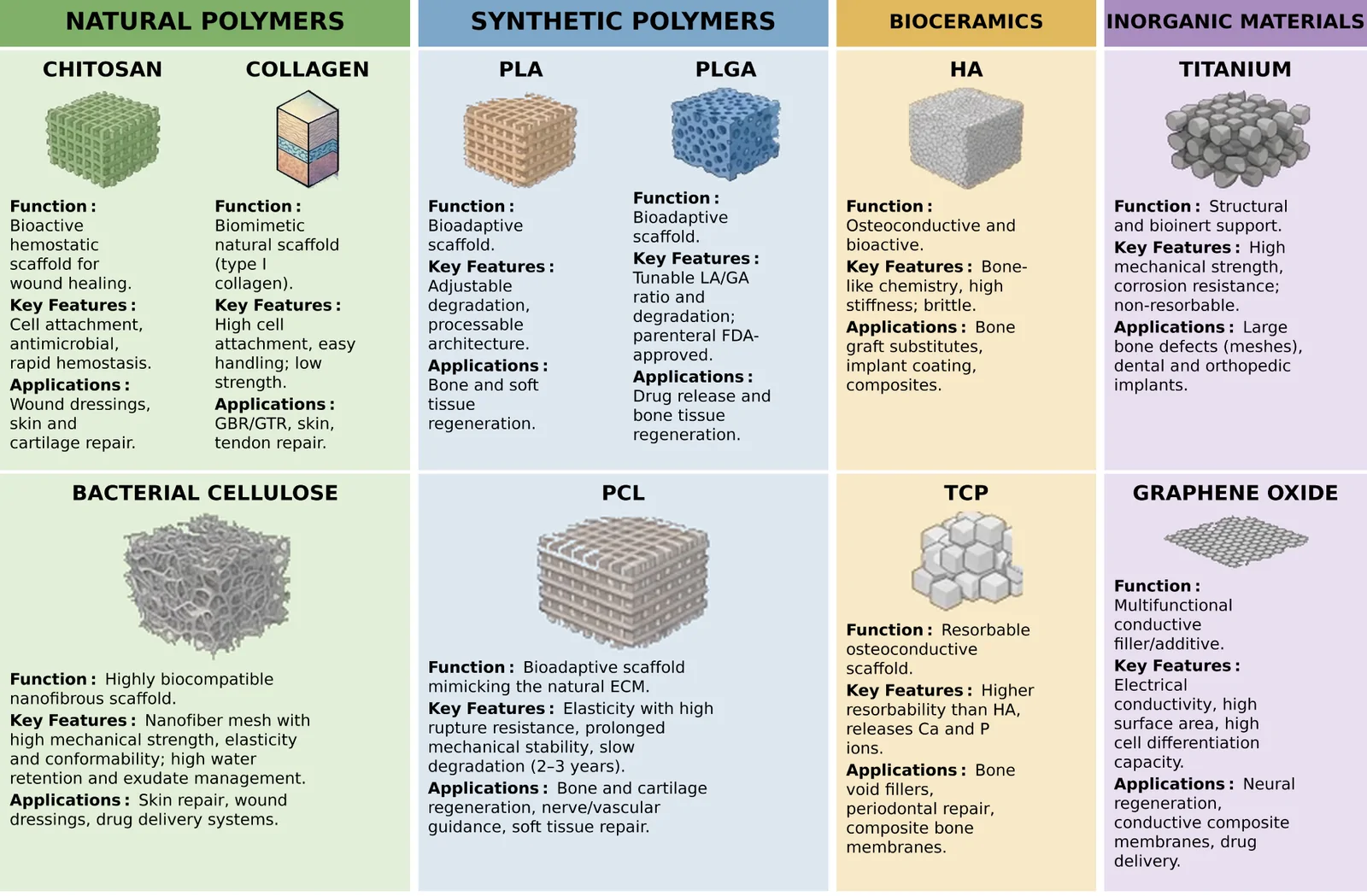
Common materials used for tissue regeneration membranes. ECM: extracellular matrix. FDA: Food and Drug Administration. GA: glycolic acid. GBR: guided bone regeneration. GTR: guided tissue regeneration. HA: hydroxyapatite. LA: lactic acid. PCL: polycaprolactone. PLA: poly­(l-lactic acid). PLGA: poly­(lactic-*co*-glycolic acid). TCP: β-tricalcium phosphate. This image was created with Google AI Studio using the Nano Banana Pro model.

### 2.1 Membrane Materials

The choice of material is the most determining factor for the biological and mechanical performance of a membrane.^22^ The ideal material will enable the creation of a scaffold with the suitable physical properties. Furthermore, its compatibility with biological-response-stimulating systems and its inherent biocompatibility will yield an efficient, personalized tissue regeneration system.

Among natural polymers, collagen (Figure 1) stands out as one of the main materials of choice in clinical practice, since it is the most abundant structural protein in human tissues.^30^ The literature reports almost 30 types of collagen in mammals, of which type I is the most abundant and employed for medical purposes.^31^ This collagen type, of bovine or porcine origin, is the basis for the most widely used membranes in bone and periodontal regeneration, exemplified by products: Bio-Gide (Geistlich Pharma), Mem-Lok (Zimmer Biomet), and Ossix Plus (Datum Dental).^30^ The popularity of collagen as a biomaterial stems from its excellent biocompatibility and low antigenicity, which enable it to be readily absorbed by the body.^32^ However, its degradation rate can be unpredictable, and the mechanical properties of pure collagen are poor.^31^ Early degradation of the collagen membrane resulting from matrix metalloproteinase activity and macrophage-mediated enzymatic processes may impair membrane stability during the initial phases of healing. This premature loss of barrier function can hinder bone regeneration by permitting soft tissue invasion and promoting an excessive inflammatory response.^30,32^ To improve mechanical strength, cross-linking the collagen-based scaffolds through physical, chemical, and biological processes was developed, which can use ultraviolet light, glutaraldehyde, or transglutaminase, among other agents.^30,31^ This process restricts molecular displacement and increases the material’s mechanical strength, thermal stability, and durability. However, it can compromise biocompatibility because the excessively rigid structure hinders proper interaction between the polymer and biological tissues.^33–35^ Additionally, physical and chemical processes can lead to collagen denaturation or potential cytotoxicity, respectively. In this context, biological cross-linkers tend to be advantageous due to their safety and nontoxic nature, as reported.^31^ However, the use of standalone collagen membranes, without supporting bone grafts, has seen a sharp decline in other medical fields due to critical shortcomings in their physical and biological properties under stress and a high likelihood of collapse at the application site. Their unpredictable degradation kinetics and the poorer tissue integration of cross-linked membranes, factors that lead to delayed vascularization and a significant increase in immunological reactions, are further reducing the use of this material.

Another natural source for membrane structuring is chitosan (Figure 1), a nontoxic, biocompatible, biodegradable cationic linear polysaccharide derived from the deacetylation of chitin, one of the most abundant biopolymers after cellulose.^36–38^ A comprehensive review of chitosan characteristics and its applications in tissue engineering has recently been published.^39^ Casimiro et al. demonstrated that chitosan-based membranes developed for skin repair did not exhibit cytotoxic effects on human fibroblast cells, and no atypical signs were observed in animals, attesting to their safety and biocompatibility.^40^ Chitosan biodegradation can occur via physical and chemical mechanisms (e.g., temperature and enzymes), with the degradation rate being adjustable by the crystallinity of the molecule (degree of deacetylation).^39^ The enzymatic hydrolysis occurs by lysozymes, which cleave the glycosidic bonds, generating low-molecular-weight chito-oligosaccharides that act as bioactive agents by modulating macrophage responses and stimulating angiogenesis during tissue repair. In the bioactivity context, chitosan is versatile, including its inherent antimicrobial properties, mainly ascribed to its molecular characteristics (degree of deacetylation and molecular weight) and its environment (pH and temperature).^39,41^ This property has been explored in several studies,^42,43^ including one that demonstrated that chitosan significantly improved the antimicrobial efficacy of chlorhexidine in wound healing, with an approximately 20-fold increase compared with the control.^44^ These results mostly come from *in vitro* assays and small-animal models, so extrapolation to clinical wound care should be cautious. The mechanical fragility of pure chitosan scaffolds is a limitation, overcome by physical or chemical processes, and blending with other polymers.^45^ Recent studies have explored chitosan composites incorporating graphene oxide to develop membranes with enhanced mechanical strength and electrical conductivity for neural regeneration.^46,47^ As with collagen, cross-linking also applies to chitosan along with the same toxicological considerations regarding residual chemicals. In this context, natural cross-linkers, such as genipin, have been gaining prominence.^45^ However, despite the considerable benefits attributed to chitosan, its clinical translation is still hindered by interbatch variability, purification complexity, and solubility and processability issues.^48^ Blends of chitosan with polymers such as poly­(vinyl alcohol) (PVA) or polylactic acid (PLA) have emerged as an alternative to overcome the processability issues associated with acidic chitosan and to facilitate the use of electrospinning in the preparation of more versatile membranes.^49^

Bacterial cellulose (e.g., synonyms: bacterial nanocellulose, microbial cellulose, microbial nanocellulose, and biocellulose) is a biocompatible polysaccharide of growing interest, produced in an ultrapure form by Gram-negative bacterial cultures, whose safety has been demonstrated by several *in vitro* and *in vivo* studies.^50,51^ The biocompatibility can be attributed to its flexibility and porosity similar to those of collagen.^52^ Bacterial cellulose consists of a nanofiber mesh with high physical and mechanical strength, resulting in scaffolds with high elasticity and conformability. In addition, it can be molded *in situ* and has a high water-holding capacity, at least 100-fold its own weight, which is particularly important for wound care, including burns.^50,53^ For these medical uses, some bacterial cellulose-based membranes are available on the market, such as Biofill, Dermafill, and Suprasorb.^51^ Despite the described advantages and clinical applications of bacterial cellulose, it lacks active signals, such as antimicrobial activity, which restricts its direct functionality, and the intrinsic hydrophilicity of the unmodified molecule can impair membrane adhesion on highly exudative wounds.^51,54^ Bacterial cellulose functionalization allows tailoring of its characteristics and performance. For instance, a bacterial cellulose-based multifunctional dressing with antibacterial and hemostatic capabilities was successfully developed using chitosan and hydroxyapatite through chemical bonding or physical loading, respectively. The authors noted significant antibacterial activity and enhanced hemostatic effectiveness compared with a commercial product.^55^ The use of chitosan or cellulose nanocrystals as fillers represents another technological alternative for preparing guided tissue regeneration membranes with improved physical properties. However, obtaining chitosan nanocrystals still presents significant scale-up challenges, which make their commercial use unlikely. Alternatively, cellulose nanocrystals, which are already produced on an industrial scale, are indeed useful materials for producing these membranes, although their specific application must be carefully considered given that these crystals are not biodegradable.^56^

Synthetic polymers, on the other hand, offer remarkable structural versatility as their main advantage. In this context, their plasticity refers to the ability to precisely adjust their molecular architecture, allowing tailored mechanical properties and biodegradation rates that adapt the materials to a wide range of applications.^57^ The most prominent group comprises resorbable aliphatic polyesters, including poly­(l-lactic acid) (PLA), poly­(glycolic acid) (PGA), PLGA, and polycaprolactone (PCL) (Figure 1). Among these polyesters, PLGA stands out due to its predictability, modifiability, and mechanical strength. It is a copolymer of PLA and PGA approved by the Food and Drug Administration (FDA), including for parenteral use.^58^ As a rule, higher PGA content leads to faster degradation, except for the 50:50 ratio of PLA/PGA, which exhibits the fastest degradation.^59^ Several well-established commercial membranes on the market are made from this polymer, such as Cytoflex Resorb (Unicare Biomedical), composed of a synthetic polylactide and polyglycolide (PLA/PGA) copolymer; Resolut Adapt (W. L. Gore & Associates), also composed of PLA/PLGA; and GC Membrane (GC Corporation, Japan), a bilayer membrane specifically formulated with PLGA for periodontal tissue guidance. The PCL polymer (Figure 1) is a valuable material due to its ability to maintain elasticity while preserving high resistance to rupture under physiological conditions. However, its slow degradation rate (over 2–3 years), hydrophobic nature, and poor cell activation that impairs cell adhesion and proliferation are drawbacks for some applications of pure PCL.^60^ In general, PCL-based blends (synthetic or natural polymers) and composites (inorganic materials) are developed to overcome these restrictions and to adjust resistance, enhancing biomedical potential.^61^ Several studies using PCL-based materials are pointed out in Krumov et al. review study, including PCL-chitosan, which enhanced scaffold hydrophilicity and facilitated cell adhesion, a fundamental issue for bone and skin tissue engineering.^60^ Regarding PCL composites, they primarily focus on enhancing mechanical properties, with calcium phosphates such as hydroxyapatite (HA), β-tricalcium phosphate (TCP), and calcium polyphosphates being common fillers for the matrix.^61^

The disadvantage associated with aliphatic polyesters is the hydrolysis of their ester bonds, releasing acidic byproducts that can lower the local pH and trigger aseptic inflammatory reactions, inhibiting tissue growth or inactivating sensitive drugs encapsulated within the matrix.^62^ Overcoming this limitation involves composite strategies that buffer the microenvironment, such as incorporating basic bioceramics (HA), using polymers with a neutralizing effect (polyphosphazenes), and other alternatives for preparing PLGA or PLGA/PLA blends and nanocomposites.^18,63,64^ The balance between the drop in pH within the PLGA matrix and the pH-lowering effect observed in the target tissue where the regeneration system is implanted is crucial for determining the safety of PLGA-based membranes. Molecular weight and the lactic acid-to-glycolic acid ratio directly determine the pH-lowering effect resulting from the polymer’s degradation process.^65^ Furthermore, the target tissue or cell type must be considered, requiring special care when the goal is to stimulate bone regeneration.

In the context of inorganic materials, porous calcium phosphate bioceramic scaffolds are considered crucial for bone tissue regeneration.^66^ HA and TCP (Figure 1) are the most extensively studied materials due to their chemical resemblance to the mineral phase of natural bone and biocompatibility. Moreover, they exhibit osteoconductivity and bioactivity, promoting cell adhesion, proliferation, and osteogenic differentiation.^66,67^ Despite this potential, pure HA and TCP are quite fragile and difficult to fabricate into the desired size and shape, and the former is nondegradable upon implantation, which restricts their broader clinical use.^67–69^ Thus, some studies focus on HA- or TCP-based composites with synthetic or natural polymers to strategically overcome the limitations of pure ceramics by leveraging their functionality and biocompatibility.^68,70^ For instance, an HA-collagen (80:20; w/w%) scaffold has been developed with appropriate flexibility, porosity, and handling properties, in which a clinical trial showed that it outperformed the gold-standard surgical implant (TCP) for bone regeneration.^71^ On the other hand, although biocompatibility is widely reported for HA and collagen separately, the associated HA-collagen scaffold resulted in a higher incidence of nonsevere adverse events relative to the TCP product.^71^ In the case of TCP, Martínez-Vázquez et al. observed that a greater flexural strength improvement resulted from its impregnation with PCL and PLA, ascribed to a more effective transfer of stress to the polymer.^66^ In addition to the benefit of mechanical properties, coating TCP matrices with polymers, such as PLGA, provides functional groups capable of anchoring molecules through covalent bonding, optimizing functionality.^72^ Furthermore, TCP can be doped with various ions, such as silver, to impart bactericidal activity to the implant site, an important concern following bone surgeries.^69,73^

Nonresorbable synthetic material membranes, such as titanium meshes and PTFE (dense or expanded), remain indispensable in cases requiring maximum space maintenance, despite the need for a second surgical procedure and the high risk of biofilm-related infections.^74,75^ These materials are among the most reliable nonresorbable biomaterials for regenerative applications because of their exceptional mechanical stability, corrosion resistance, and biocompatibility.^75,76^ Moreover, they are particularly valuable in guided bone regeneration (GBR) because they maintain regenerative space, resist collapse under soft-tissue pressure, and support large-volume bone augmentation procedures.^77^ For very large malformations, titanium meshes, such as those from the Pro-Fix line (DePuy Synthes), offer the maximum rigidity. Dense PTFE (d-PTFE) membranes have an essentially nonporous microstructure, offering superior resistance to bacterial penetration and soft tissue migration. For example, the high d-PTFE commercial membrane cytoplast (Osteogenics Biomedical) was developed to resist bacterial permeation in cases of oral exposure. Conversely, expanded PTFE (e-PTFE) membranes exhibit an interconnected fibrillar porous network that favors tissue integration and nutrient exchange, and are more susceptible to microbial colonization. Therefore, d-PTFE is generally preferred when enhanced barrier function is required, whereas e-PTFE may provide advantages in regenerative procedures where tissue interaction is desirable.^75^

Graphene is a carbon-based flat monolayer, which has emerged as an attractive material for regenerative tissue membrane development because of its robust structure, unique mechanical strength, high surface area, electrical conductivity, thermal stability, and biocompatibility.^78^ Electrical conductivity supports cellular communication and mineralization, both crucial for effective bone healing.^79^ The graphene family of nanomaterials includes graphene oxide and graphene-based composites (Figure 1), the latter resulting from graphene functionalization.^80^ The two-dimensional lamellar structure of graphene oxide, with functional groups on its surface (hydroxyl, carboxyl, and carbonyl), provides a hydrophilic character.^81^ Graphene oxide membranes have demonstrated *in vitro*, improved cellular attachment, stem cell adhesion, and differentiation, which is particularly promising for bone, neural, and skin tissue engineering.^80^ In bone applications, for instance, graphene oxide substrates may favor osteogenic commitment, effects attributed to the combination of electrical conductivity, surface chemistry, and nanoscale topography.^78,80^ Additionally, graphene oxide is easy to functionalize with polymers and small molecules, thereby improving or modifying properties.^80^ This ease of functionalization has been explored, for example, in chitosan/xanthan and PLA matrices, in which graphene oxide acts simultaneously as mechanical reinforcement and conductive filler, yielding composite membranes with properties not attainable by the polymers alone.^47,78^ In this context, a recent systematic review highlighted composites of graphene oxide-PLA and graphene oxide-HA as the most promising for clinical trials focused on bone regeneration in dentistry.^78^

A comparative description of the main characteristics of membrane materials previously presented is summarized in Table 1.

**Table 1:** Membrane Material Characteristics: A Comparative Evaluation^a^

Material	Biodegradation rate	Mechanical Strength	Porosity	Bioactivity	Main Advantage	References
Collagen	+	-	±	+	Biomimicry	^30,31^
Chitosan	±	-	±	+	Antimicrobial activity	^37–39^
Bacterial cellulose	±	+	+	+	High water retention	^50–52^
PLA	±	+	Adjustable	-	Tailorable processing and architecture	^60^
PLGA	±	±	Adjustable	±	Adjustable degradation rate	^58,59^
PCL	-	+	+	±	Prolonged mechanical stability	^60,61^
HA	-	-	±	+	Bone-like chemistry and osteoconductivity	^66,67^
TCP	+	-	±	+	Resorbable osteoconductive ceramic	^66,68^
Titanium mesh	Nonbiodegradable	+++	Not applicable	-	High structural capacity	^75^
PTFE	Nonbiodegradable	++	-	-	High regenerative space	^74,75^
Graphene oxide	Variable	±	Adjustable	++	Electrical conduction	^78,80^

a Low: (−); moderate: (±); high: (+); very high: (++); and extremely high: (+++). HA: hydroxyapatite. PCL: polycaprolactone. PLA: poly­(L-lactic acid). PLGA: poly­(lactic-*co*-glycolic acid). PTFE: polytetrafluoroethylene. TCP: β-tricalcium phosphate.

Overall, a comparative analysis of different membrane types is fundamental to understanding their potential and limitations for therapeutic applications. As pointed out, each material presents specific characteristics that influence biological performance, cellular response, and durability in a physiological environment, limiting the possibility of having a single material that meets all clinical needs.^32,82,83^ Natural membranes offer superior biocompatibility but also exhibit structural fragility and major challenges for scale-up and, consequently, the attainment of commercial products.^32^ Synthetic membranes allow greater control over mechanical properties and degradation rates, although they can induce unwanted inflammatory responses in some situations.^64^ Within this category, high-density materials such as e-PTFE and titanium meshes stand out for their rigidity, which is necessary to maintain space in large areas. However, their nonresorbable nature necessitates a second surgical intervention for removal.^74^ In this context, composites emerge as a promising alternative by combining complementary properties and allowing fine performance adjustments,^21,84^ indicating that the future evolution of the field depends on the development of hybrid biomaterials capable of uniting mechanical stability, biological functionality, and clinical safety in a single therapeutic platform.^47,85^

In our view, the practical question is not which material is best, but which limitation is least harmful for each application: collagen remains unmatched where handling and rapid integration are decisive, PLGA where degradation must be programmed, PCL where prolonged mechanical support is required, bacterial cellulose where exudate management dominates, and graphene-based composites where electrical cues are desired. In quantitative terms, reported degradation windows span from a few weeks for non-cross-linked collagen and 50:50 PLGA to months for PLA and 2–3 years for PCL, while tensile strength increases from the low megapascal range of hydrated natural polymers to values one to two orders of magnitude higher for PCL, PTFE, and titanium meshes.^30,59,60,74^

Regarding material applications and clinical evidence (Figure 1), collagen membranes have the strongest randomized-trial support in GBR/GTR and are the routine first choice, despite their rapid degradation and low mechanical strength for load bearing. For large or noncontained defects, e-PTFE and titanium meshes are clinically established materials, although they require removal surgery and higher exposure-related complications. Resorbable synthetic polymers have clinical support in selected applications for long-term use because of their controlled degradation rate, with inflammatory reactions as the main complication. Despite their promise, especially in terms of functionality, bacterial cellulose and graphene oxide remain essentially at the preclinical stage. Despite their promise, especially in terms of functionality, graphene oxide-based membranes remain at the preclinical stage, whereas bacterial cellulose, although commercially available as a wound dressing, still lacks robust clinical trial evidence for regenerative indications, and its functionalized variants remain preclinical.

### 2.2 Membrane Morphology

Regarding morphology, the three-dimensional organization of the membrane, from the macro- to the microscale, directly influences the biological response. The most fundamental morphological classification distinguishes membranes into dense and porous (Figure 2). Dense membranes, such as polymeric films without transmural porosity, function as totally occlusive barriers. Although this occlusivity effectively prevents the penetration of bacteria and nontarget cell types, it also hinders the transport of fluids, nutrients, and oxygen, potentially leading to necrosis of the underlying tissue and thus severely limiting its clinical application.^86^ For this reason, most membranes for biological tissue engineering are designed to be porous. Porosity, defined as the volume fraction of voids in a solid, and pore interconnectivity are vital to allow the perfusion of body fluids, cell migration into the lesion, and, crucially, neovascularization, which is indispensable for the maintenance and integration of any regenerated tissue.^87,88^

**Figure 2:**
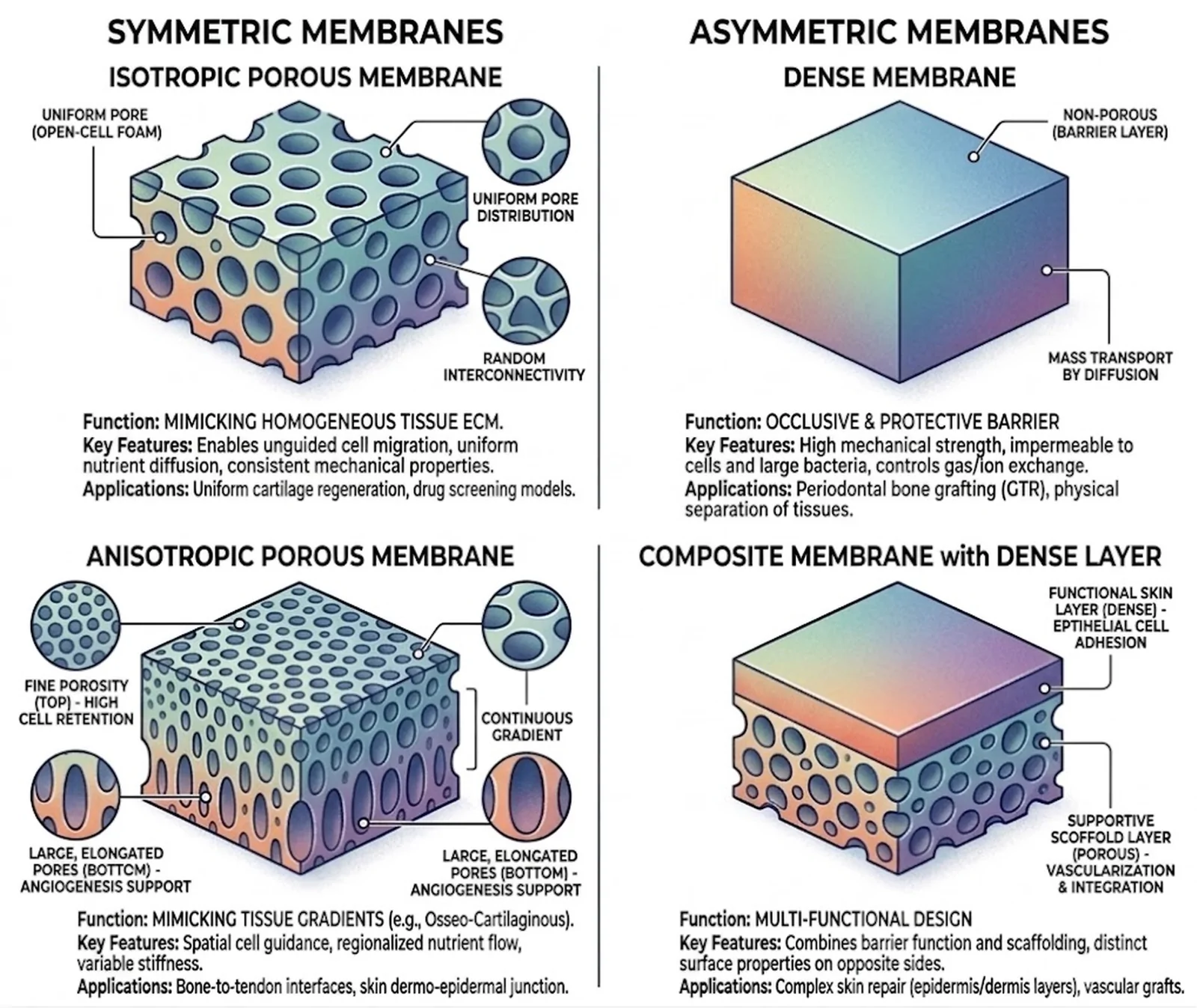
Schematic diagram of membrane types. Typical pore size for tissue regeneration: skin 2–100 μm; bone 100–600 μm; cardiac 25–60 μm; and alveolar 20–100 μm.^89^ ECM: extracellular matrix. This image was created with Google AI Studio using the Nano Banana Pro model.

The current debate focuses on optimizing pore morphology to meet the specific cellular demands of each tissue. For bone regeneration, it has been established that pores in the range of 100–600 μm are ideal for allowing osteoblast migration and the formation of robust blood vessels, while providing satisfactory mechanical support.^83,89^ In contrast, applications in skin tissue engineering benefit from matrices with graded porosity, where smaller pores, between 2 and 100 μm, favor fibroblast adhesion and dermal regeneration without compromising the barrier against fluid loss.^89^ The literature shows that multilayered matrices combining regions of different porosities outperform those with uniform pores by mimicking the hierarchical architecture of native tissues. In addition to porosity, spatial organization determines whether the membrane is symmetric or asymmetric,^29,90^ as illustrated in Figure 2. Symmetric membranes have a uniform pore distribution, whereas asymmetric or gradient membranes offer a more sophisticated approach that mimics tissue complexity. For example, a GBR membrane benefits from an anisotropic architecture with a dense outer layer to serve as a bacterial barrier and a macroporous inner face to promote bone integration.^29^

Microarchitecture and surface chemistry determine immediate biological interactions, governed by protein adsorption and the Vroman effect. As soon as the biomaterial interacts with blood, its surface is covered by a dynamic protein corona whose conformation dictates the implant’s fate. The favorable adsorption of adhesive proteins such as fibronectin and vitronectin exposes binding domains, such as RGD, that facilitate cell adhesion via integrins, promoting tissue integration. Conversely, if the surface induces denaturation of these proteins or promotes fibrinogen accumulation, the immune system may recognize the material as a threat, triggering a chronic inflammatory response and fibrous encapsulation. Therefore, controlling wettability and surface charge aims to modulate the adsorbed layer to ensure it acts as a signaling bridge for regeneration rather than a trigger for rejection.^91,92^ Adjusting process parameters and determining their scalability will establish an optimal balance between porosity, which ensures the effectiveness of the barrier function and controls cell migration, and roughness, which influences cell adhesion and angiogenesis. This balance is a key factor that often determines the clinical failure of membranes developed at the laboratory scale.

### 2.3 Membrane Manufacturing

The materialization of the various discussed morphologies and mechanical properties is achieved through specific manufacturing techniques, each conferring distinct physicochemical and biological properties on the biomaterial. The choice of manufacturing method is decisive, as it determines the degree of porosity, pore interconnectivity, mechanical strength, roughness, and surface topography of the membrane, factors that directly influence the cellular response. Table 2 presents a critical comparison of the most widely used tissue engineering techniques, highlighting their operating principles and practical implications for clinical use, as described in the literature. For instance, the phase inversion technique is one of the most robust and scalable techniques for producing porous membranes. In this context, the Loeb-Sourirajan process is a classic industrial example that employs the precipitation of a polymer solution to form anisotropic membranes, constituting the technological basis for the manufacture of reverse osmosis filters and polysulfone hemodialysis membranes widely used in clinical practice. However, reliance on toxic organic solvents such as dimethylformamide (DMF) poses environmental and biosafety risks. In response, the industry has adopted green chemistry principles, substituting these agents with sustainable, biomass-derived solvents such as gamma-valerolactone for processing polymers like PLA, thereby reducing the device’s carbon footprint and residual toxicity. Alternatively, lyophilization eliminates the need for harsh solvents, thereby producing highly porous matrices. To overcome the limitation of pore irregularity, the technique is combined with the use of porogens. The addition of sodium chloride particles with controlled granulometry (100–250 μm) to the polymer solution allows the achievement, after washing, of predefined pore dimensions and high interconnectivity, which are essential for cell migration, in contrast to the random morphology obtained by conventional lyophilization. A limitation of this technique would be the requirement for water solubility of the materials used and the cost of an industrial plant utilizing freeze-dryers.

**Table 2:** Techniques, Theoretical Basis, Advantages, and Limitations for Obtaining Membranes

Technique	Theoretical basis	Advantages	Limitations	References
**Phase Inversion and Solvent Casting**	Deposition of the polymer solution onto a support followed by solidification via immersion in a coagulation bath (phase inversion) or solvent evaporation (solvent casting).	Robust and industrially scalable technique, allowing the production of high-flux and asymmetric membranes. Evaporation casting allows the formation of dense and occlusive films.	The use of toxic organic solvents such as dimethylformamide requires rigorous control and exhaustive washing processes to avoid residual cytotoxicity. The search for “green” solvents is an ongoing challenge.	^10,97,98^
**Lyophilization (Freeze-drying)**	Freezing of a polymer solution or hydrogel followed by solvent (usually water) sublimation under vacuum, creating a porous structure where ice crystals previously existed.	Operational simplicity and absence of harsh organic solvents, resulting in matrices with high porosity and favorable interconnectivity for cellular infiltration.	Limited control over pore morphology, which tends to be irregular unless directional freezing techniques or porogens are used. Time-consuming and high-energy consumption process.	^3,99^
**Electrospinning**	Application of high electrical voltage to a polymer solution to draw a fluid jet that solidifies, forming nanofibers deposited on a grounded collector.	Produces fibrillar membranes with high surface area that mimic the architecture of the native extracellular matrix, being ideal for cell adhesion and drug release.	Difficulty in creating thick three-dimensional structures (usually results in 2D mats) and low industrial production rate, although melt electrospinning mitigates solvent use.	^17,100^
**3D Printing and Bioprinting**	Additive manufacturing that deposits material layer by layer based on a digital model. Bioprinting uses “bioinks” containing living cells.	Precise control of internal and external architecture, allowing anatomical customization (precision medicine) and the fabrication of complex structures with porosity gradients.	Limited resolution compared to electrospinning. Bioprinting faces challenges in postprocessing cell viability and the need for vascularization for thick constructs.	^93,94,101^

3D printing and bioprinting represent the vanguard of personalized membrane synthesis. Unlike standardized commercial membranes that require intraoperative cutting and adaptation, additive manufacturing allows the fabrication of barriers with exact anatomical geometry based on the patient’s imaging exams, ensuring a perfect seal in complex defects, minimizing variability in patient responses.^83^ In bioprinting, bioinks composed of gelatin or alginate hydrogels loaded with living cells enable the creation of skin substitutes and biomimetic periosteal membranes. As pointed out by Farjaminejad et al., the incorporation of cells directly into the bioink overcomes one of the main limitations of the technique, which is the nonhomogeneous distribution of the cells in the printed membrane.^83^ In addition, the current challenge focuses on developing materials that are simultaneously printable and capable of maintaining postprocessing cell viability, but the technology already proves viable for the production of bioactive dressings that release growth factors in a spatially controlled manner.^93,94^ The major constraint on the use of 3D printing, beyond adjusting the rheological properties of the mixture to be printed, is the preferential use of hydrogels in this technique, along with the difficulties in tuning the porosity and stiffness of the resulting membranes and the limitations regarding the use of thermosensitive materials.

The electrospinning technique stands out for producing nanofiber mats that mimic the physical architecture of ECM. Although the low production rate is a bottleneck, advances such as melt electrospinning and the use of multiple emitters have made it scalable. Electrospinning fabricates fibrous materials with controlled micro- and nanoscale architectures for applications across various technological fields. For example, in the biomedical field, while solution-electrospun mats often yield dense structures with small pores that limit cellular infiltration, melt-electrospinning serves as a direct writing technique. This method deposits fibers layer by layer to construct 3D scaffolds with open interconnected pore networks designed to promote cellular invasion and tissue regeneration. In dentistry, this capability is used to fabricate a fibrous membrane that constitutes a multiphasic scaffold for periodontal-ligament regeneration. Other complex structures, such as tubular vascular grafts and humanized bone models for studying cancer metastasis, are also manufactured with this approach.^17,95^ Considering membranes as drug delivery systems, the high surface area of the fibers accelerates the dissolution of poorly water-soluble drugs. The coaxial melt electrospinning technique creates fibers with a core–shell structure, in which an active ingredient is encapsulated within a polymeric shell to enable controlled, sustained release. This technique also fabricates sensors, such as thermochromic fibers that encapsulate a phase-change material in their cores, causing the fibers to change color in response to body temperature.^96^ With the development of industrially applicable processes and equipment, the electrospinning technique stands out as the most promising among the techniques described.

## 3 Clinical Applications of Tissue Regeneration Membranes

### 3.1 Guided Bone and Periodontal Regeneration

The principle of guided regeneration is one of the most successful and widely studied applications of membrane technology in medicine and dentistry, involving modulation of cellular competition during the healing of bone or periodontal defects.^102,103^ As demonstrated in the pioneering studies by Nyman et al., epithelial cells and adjacent connective tissue cells present a higher proliferation and migration rate than osteogenic and periodontal ligament cells, so that, in the absence of a barrier, these fast-growing cell populations occupy the lesion space, preventing the desired tissue regeneration and resulting in a predominantly fibrous repair.^102^ To mitigate this competition, the GBR or guided periodontal regeneration (GPR) membrane is positioned as a mechanical and occlusive barrier, isolating the defect from the overlying soft tissue, which creates a protected and stable compartment for the organization of the blood clot and subsequent repopulation by cell types with regenerative potential, as described by Elgali et al.^13^ Currently, the gold standard for most GBR and GPR applications is the use of resorbable collagen-based membranes, frequently combined with bone graft materials to provide mechanical support to the clot and prevent space collapse, with high long-term implant survival rates, consolidating it as a safe and predictable procedure for clinical practice.^15,26^ This approach, as noted in the studies by Bottino et al. and Ren et al. has surpassed the use of nonresorbable e-PTFE and titanium membranes, primarily because it eliminates the need for a second surgical intervention to remove them, thereby significantly reducing patient morbidity.^15,26^ Despite the success indicated by this high survival rate in small- to moderate-volume bone defects, the current scientific debate focuses on overcoming the limitations of the technique in more extensive defects, where conventional collagen membranes may be insufficiently stiff to maintain space.^104^ Additionally, the management of complications, such as premature membrane exposure, poses a significant clinical challenge, as discussed by Das et al., who investigate strategies to optimize flap stability and soft tissue healing. Membrane-assisted tissue engineering has also been proposed beyond periodontal and peri-implant defects, including strategies to accelerate bone remodeling and reduce treatment time in orthodontic and orthognathic therapy.^105^

In an analogous field, orthopedics presents the Masquelet technique as an effective biological alternative for treating critical bone defects.^106^ The procedure detailed by Das consists of the insertion of a bone cement spacer at the defect site to induce the formation of an autologous synovial membrane, which is highly vascularized and rich in growth factors such as VEGF and TGF-β1, creating a biologically optimized environment to receive the bone graft in a second stage.^104^ Subsequent studies indicate that a 2- to 6-week interval between surgical stages provides the highest angiogenic and osteogenic potential of the induced membrane, favoring better outcomes in complex cases associated with trauma and osteomyelitis.^106^ The use of resorbable collagen and nonresorbable membranes in GBR and GPR is supported by randomized clinical trials, systematic reviews, and decades of routine clinical practice, whereas most bioactive, third-generation membranes remain at the preclinical stage.

### 3.2 Cartilage Engineering

Articular cartilage, a tissue with an extremely limited intrinsic healing capacity due to its avascular nature and low cellularity, presents a significant clinical challenge when injured. Focal chondral lesions, common in young and active populations, frequently progress to degenerative osteoarthritis and chronic pain if not properly treated, underlining the need for effective interventions.^107,108^ Early treatment attempts, such as the microfracture technique, aimed to stimulate repair by recruiting bone marrow cells but often resulted in fibrocartilage that was mechanically inferior and had limited durability. This scenario motivated the development of cell therapies, such as autologous chondrocyte implantation (ACI).^109,110^ The natural evolution of ACI led to the development of matrix-induced autologous chondrocyte implantation (MACI), in which 3D membranes became a central and indispensable component. In this technique, the membrane serves as a framework or scaffold that transports and confines the autologous chondrocytes, previously expanded in the laboratory, directly to the lesion site. These membranes, generally composed of biocompatible and biodegradable materials such as type I/III porcine collagen or hyaluronic acid derivatives, provide an essential substrate for adhesion, proliferation, and deposition of new extracellular matrix by the chondrocytes.^110,111^

Commercial products such as MACI (Vericel Corporation) use a type I/III porcine collagen membrane seeded with autologous chondrocytes, while Chondro-Gide (Geistlich Pharma) is a double-layer collagen membrane designed with a smooth face to prevent soft tissue adhesions and a porous face to facilitate cellular integration.^110,112^ Clinical studies and systematic reviews have shown durable long-term outcomes of MACI, with advantages over microfracture particularly for larger lesions, resulting in better clinical outcomes, such as pain reduction and functional improvement, and the formation of repair tissue with characteristics closer to native hyaline cartilage.^108,112^ Despite clinical success, the MACI technique still faces significant limitations, including high cost, the need for two distinct surgical procedures (biopsy and implantation), and the risk of chondrocyte dedifferentiation during *in vitro* cell expansion. This scenario has motivated the research frontier to focus on developing single-stage and “off-the-shelf” strategies. The current trend is toward bioactive acellular membranes functionalized with biological signals, such as peptides or growth factors, to recruit the patient’s own stem cells from the subchondral bone and guide them into chondrogenic differentiation directly at the lesion site. This approach, known as *in situ* chondrogenesis, represents a promising evolution by simplifying the procedure and eliminating the need for cell culture.^108,110,112^

Reinforcing the centrality and efficacy of membranes as carrier platforms, Palhares et al. reported excellent functional outcomes in patients 2 years after the treatment of chondral lesions in the patella. This clinical study supports the robustness of the membrane as a critical component for the success of cellular therapies, ensuring the distribution and maintenance of cells in the complex joint environment, thereby translating into significant functional recovery for patients.^113^ Cell-seeded collagen or hyaluronic acid membranes (MACI) are supported by comparative clinical trials and systematic reviews, whereas acellular in situ chondrogenesis strategies remain restricted to in vitro and animal studies.

### 3.3 Skin Regeneration

Extensive skin loss, resulting from conditions such as severe burns or chronic ulcers, notably diabetic foot ulcers, compromises the integument’s barrier function, exposing the body to significant risks of infection, dehydration, and electrolyte imbalance. In this context, skin substitutes and advanced dressings are essential for temporarily mimicking the epidermal barrier and, ideally, providing a scaffold for dermal regeneration.^114^ Membranes formulated from natural polymers, such as collagen, chitosan, and alginate, are widely used for their excellent biocompatibility and ability to maintain a moist wound bed, a fundamental condition for optimizing the healing process, as noted in the 2023 studies by Vecin and Kirsner.^115^ These membranes, by their nature, promote cell adhesion and proliferation, serving as a basis for tissue reconstruction.^115^ The most successful approach for full-thickness wounds involves using dermal regeneration templates, such as the Integra Dermal Regeneration Template system (Integra LifeSciences), approved by the FDA for third-degree burns in 1996 and for diabetic foot ulcers in 2016. This double-layer device consists of a porous matrix of collagen and glycosaminoglycans that serves as a scaffold for infiltration of the patient fibroblasts and blood vessels, forming a functional “neodermis” over several weeks. During this period, an upper silicone layer protects the wound and acts as a temporary epidermal barrier. Once the neodermis is vascularized, the silicone layer is removed, and a thin split-thickness skin graft from the patient is applied to definitively close the wound.^116^ The field of advanced dressings is moving beyond passive coverage, seeking to incorporate functionalities that transform them into active therapeutic platforms. These smart matrices are frequently designed to be triggered by environmental stimuli intrinsic to the wound, such as pH variations, oxygen gradients, and temperature fluctuations, or by integrating flexible microsensors that monitor the lesion’s status in real time.^116^

One of the major challenges in managing chronic wounds is bacterial infection, which delays or prevents healing; in response, recent research has focused on the development of functional membranes. For instance, recent research highlights the development of functional dressings based on responsive hydrogels and sensing materials, as well as chitosan- and bacterial cellulose-based membranes loaded with silver or zinc oxide nanoparticles.^117–119^ As reviewed by Alven and Aderibigbe, these membranes exhibit potent, sustained-release antimicrobial activity, effectively controlling the bacterial load in the wound bed without inducing significant cytotoxicity in host cells.^120^ In the context of smart dressings, classified at the frontier of innovation, they also integrate diagnostic capabilities. Han et al. describe the development of sensor membranes incorporating colorimetric indicators that change color in response to local pH shifts or to the presence of specific enzymes released by pathogenic bacteria, such as *Pseudomonas aeruginosa* elastase.^118^ While currently confined to laboratory settings, this color change provides a visual, noninvasive, real-time diagnosis of the infection, enabling faster, more targeted clinical intervention before the infection establishes itself. Such systems represent a significant advance toward personalized medicine in wound treatment, although their clinical translation is hindered by the lack of robust human trials confirming safety, scalability, and long-term efficacy.^118^

When comparing clinical indications and material properties, natural materials are taking center stage in skin regeneration given the highly dynamic, vascularized, and immunologically active nature of cutaneous tissue. Unlike bone tissue, where the mechanical rigidity of a synthetic polymer (or cross-linked collagen) is often desirable to maintain physical space (e.g., GBR), skin requires immediate bioactivity and elastomeric flexibility. This fact does not entirely rule out synthetic materials, which can even be combined with natural materials for skin regeneration to modulate the degradation rates. Natural polymer dressings and dermal regeneration templates are supported by clinical trials and established clinical use, whereas nanoparticle-loaded and sensor-integrated smart dressings are currently supported by *in vitro* and animal data only.

### 3.4 Neural Tissue Engineering

The regeneration of peripheral nerves after injuries that result in substance loss and spacing between nerve stumps constitutes a complex surgical challenge. Although the autologous graft remains the gold standard, its use is limited by donor site morbidity and scarcity of available material. In this scenario, neural guidance conduits (NGCs) emerge as an effective alternative.^3,28^ An ideal NGC must present tailored biodegradability and selective permeability, allowing the diffusion of nutrients while blocking the infiltration of fibrous scar tissue, serving as a tunnel to direct axonal growth from the proximal to the distal stump.^121^ Clinical evidence indicates that, for short gaps (generally <3 cm), commercial collagen conduits such as NeuraGen offer functional recovery comparable to that of autologous grafts, whereas porcine ECM connectors such as Axoguard are indicated for gaps of up to 5 mm; adjuvant cell-based strategies to extend these limits remain at the preclinical stage.^122,123^ However, the regeneration of large defects still presents failure due to axonal dispersion. To overcome this barrier, the new generation of conduits incorporates internal topography with aligned fibers that provide physical cues for cellular orientation and electrical bioactivity strategies.^124^ Membranes composed of conductive polymers or nanomaterials, such as MXenes and graphene, have demonstrated the ability to deliver localized electrical stimulation, accelerating Schwann cell proliferation and axonal elongation and thereby transforming passive guidance into active regenerative stimulation.^125^ Despite promising preclinical results, evidence of nanomaterial-based NGCs remains limited. Direct clinical comparisons are nonexistent, and the potential long-term neurotoxicity and unpredictable degradation profiles of these advanced materials represent severe limitations that must be addressed before human application. Membranes in dentistry, specifically in implantology and periodontics, are a well-established, routine application, supported by strong clinical evidence. Less common uses include sodium hyaluronate/carboxymethylcellulose films (e.g., Seprafilm) following extensive laparotomies to prevent adhesion as well as in abdominal, gynecological, and tendon surgeries. For most other applications, such as orthopedics, nerve regeneration, and vascular grafts, early clinical studies and basic research are underway; however, in daily surgical practice, these applications encounter insurmountable biological limitations, remaining confined to specific niches. Clinical evidence for nerve guidance conduits is restricted to short nerve gaps (<3 cm) and derives mainly from comparative clinical studies against autologous grafts, whereas conductive and nanomaterial-based conduits have been evaluated in animal models only.

To summarize the potential clinical uses and the level of technological maturity, Table 3 provides a critical comparative analysis of the discussed membranes, highlighting their primary indications, inherent limitations, and current levels of evidence.

**Table 3:** Comparative Analysis and Clinical Indications of Tissue Regeneration Membranes^a^

Tissue Application	Membrane Type/Material	Primary Clinical Indication	Key Advantages	Critical Limitations	Level of Evidence
Bone and Periodontal	**Resorbable Collagen** (often with bone grafts)	Small to moderate-volume bone or periodontal defects.	Eliminates second surgery for removal; high biocompatibility; high long-term implant survival	Insufficient stiffness for extensive/vertical defects; rapid enzymatic degradation and loss of barrier function upon premature exposure.	**High** (routine clinical practice supported by randomized clinical trials)^15,26,104^
Bone and Periodontal	**Nonresorbable** (d-PTFE, e-PTFE, titanium mesh, titanium-reinforced PTFE)	Extensive or vertical bone defects requiring space maintenance and large-volume augmentation.	High structural rigidity; excellent mechanical barrier function.	Requires a second surgical intervention for removal, increasing patient morbidity; higher risk of exposure and biofilm-related infection.	**High** (long-standing clinical use; randomized trials and systematic reviews)^26,74–77^
Cartilage	**Cell-seeded collagen I/III or hyaluronic acid membranes** (MACI)	Focal chondral lesions, particularly larger-diameter defects.	Provides essential substrate for chondrocyte adhesion; yields tissue closer to native hyaline cartilage.	High cost; requires two surgical procedures (biopsy and implantation); risk of cell dedifferentiation.	**High** (FDA-approved therapy; randomized trials versus microfracture, systematic reviews and long-term clinical series)^110–113^
Cartilage	**Bioactive acellular** (functionalized with peptides/growth factors)	Investigational: single-stage, “off-the-shelf” in situ chondrogenesis.	Eliminates cell culture steps and a second surgery by recruiting endogenous cells.	Long-term mechanical stability and efficacy in human joints remain unknown.	**Low** (*in vitro* and animal models only)^110^
Skin	**Natural polymers** (collagen, alginate, bacterial cellulose, chitosan)	Superficial wounds and exuding ulcers.	Maintains a moist wound bed; immediate bioactivity and flexibility.	Lacks mechanical scaffolding required for full-thickness reconstruction.	**High for collagen, alginate, and bacterial cellulose dressings** (established clinical use); **low to moderate for chitosan-based membranes** (mainly *in vitro* and small-animal data)^40,44,51,115^
Skin	**Dermal regeneration templates** (collagen-GAG + silicone, e.g., Integra) **and acellular dermal matrices**	Extensive, full-thickness skin loss (severe burns, diabetic foot ulcers).	Provides a functional “neodermis” scaffold; temporary epidermal barrier.	Requires a subsequent split-thickness skin graft for definitive closure.	**High** (FDA-approved for burns since 1996 and diabetic foot ulcers since 2016; established clinical use)^116^
Skin	**Smart/functionalized** (nanoparticles, colorimetric sensors)	Investigational: chronic or infected wounds requiring antimicrobial control and real-time monitoring.	Targeted antimicrobial release; noninvasive visual diagnosis of infection markers.	Lack of robust human trials confirming safety, scalability, and long-term efficacy.	**Low** (*in vitro* and animal studies only)^117,118,120^
Neural	**Standard nerve guidance conduits** (NGCs; collagen, porcine ECM)	Short peripheral nerve gaps (collagen conduits: <3 cm; porcine ECM connectors: ≤5 mm).	Avoids donor site morbidity of autologous grafts; selective permeability.	High clinical failure rate in large defects (>3 cm) due to axonal dispersion.	**Moderate to high** (clinical evidence restricted to gaps <3 cm)^121,123,124^
Neural	**Conductive/nanomaterial NGCs** (MXenes, graphene)	Investigational: long-gap nerve defects requiring active electrical stimulation and topographical cues.	Delivers localized electrical stimulation to accelerate Schwann cell proliferation.	Potential long-term neurotoxicity; unpredictable degradation; direct clinical comparisons do not exist.	**Low** (*in vitro* and animal models only)^124,125^

a Level of evidence (qualitative hierarchy adopted in this review): high, supported by randomized clinical trials, systematic reviews, or long-standing routine clinical use; moderate, supported by clinical case series or by trials restricted to specific indications; low, supported by *in vitro* and/or animal studies only, without clinical trials. Superscript numbers refer to the reference list. ECM: extracellular matrix. GAG: glycosaminoglycan. MACI: matrix-induced autologous chondrocyte implantation. NGC: nerve guidance conduit. PTFE: polytetrafluoroethylene (d, dense; e, expanded). FDA: Food and Drug Administration.

From a clinical decision-making standpoint, Table 3 indicates that membrane selection should be driven primarily by the mechanical demand of the defect and by the maturity of the supporting evidence. Resorbable collagen membranes remain the first choice for contained bone and periodontal defects because they combine the strongest clinical evidence with the lowest morbidity, whereas nonresorbable membranes are justified only when space maintenance cannot be achieved otherwise, at the cost of a second surgery and a higher exposure risk. For large focal chondral lesions, cell-seeded collagen or hyaluronic acid membranes are preferable to microfracture, while acellular alternatives are not yet ready for clinical use. In skin regeneration, natural polymer dressings are sufficient for superficial wounds, whereas dermal regeneration templates are indicated for full-thickness loss. Finally, standard conduits should be reserved for nerve gaps below 3 cm since no membrane has yet demonstrated clinical efficacy for longer gaps. Smart, conductive, and nanomaterial-based membranes, although conceptually attractive, cannot currently be recommended for any clinical indications.

## 4 New Trends and Perspectives in Membranes for Tissue Regeneration

Unless otherwise stated, the technologies discussed in this section are supported by *in vitro* and animal studies only and have not yet been evaluated in clinical trials; their clinical potential should therefore be regarded as prospective, rather than established.

### 4.1 Smart and Stimuli-Responsive Membranes

Beyond the paradigm of static devices, the current frontier of bioengineering focuses on developing smart membranes that adapt their physicochemical properties in response to fluctuations in the physiological microenvironment. This responsiveness allows for personalized and “on-demand” therapy.^126^ The range of stimuli-responsive polymers extends beyond the commonly highlighted pH- and temperature-sensitive systems in tissue regeneration. Materials responsive to light, redox potential, electric and magnetic fields, ionic strength, biomolecules, and multistimuli combinations have been reported, suggesting that much of this chemical repertoire remains to be explored as membrane platforms for tissue regeneration.^127,128^

One of the most promising strategies involves the use of pH-responsive polymers designed to release antimicrobial or anti-inflammatory agents only when they detect the local acidification characteristic of infections or exacerbated inflammation, minimizing systemic drug exposure.^129^ Another prominent class is thermoresponsive materials, exemplified by poly­(*N*-isopropylacrylamide) hydrogels, which exhibit a sol–gel phase transition at a critical temperature near body temperature. This property allows them to be injected in liquid form, perfectly filling irregular defects and solidifying *in situ* to form a stable membrane, optimizing the implant-tissue interface.^119^ Advancing toward systems with greater biological specificity, research has focused on enzyme-responsive membranes incorporating peptide bonds susceptible to cleavage by matrix metalloproteinases, which are overexpressed in tissue-remodeling microenvironments. As detailed by Minehan and Del Borgo (2022), this mechanism ensures that matrix degradation or drug release occurs synchronously with inflammatory cellular activity, ensuring that structural support is removed only as new tissue is formed.^130^ Despite their undeniable potential, clinical translation faces the challenge of validating these dynamic systems *in vivo*, where multiple parameters fluctuate simultaneously. To overcome this barrier, future perspectives point to the development of multiresponsive systems. Zelzer and Ulijn described logic platforms that integrate temperature and pH responses sequentially, allowing for refined therapeutic control that adapts to the distinct phases of the regenerative cascade.^131^

Bone is a naturally piezoelectric tissue, generating electrical potentials under mechanical stress that dictate remodeling (Wolff’s law). Consequently, electro-responsive and piezoelectric polymers, such as PLA and polyvinylidene fluoride (PVDF), are being extensively investigated for GBR applications. These smart membranes harvest mechanical energy and convert it into localized microcurrents. A notable study reported the fabrication of a biodegradable piezoelectric Janus membrane composed of electrospun PLA and gelatin. By annealing and polarizing the membrane, a specifically charged surface architecture was created. The resulting piezoelectric output not only exhibited broad-spectrum antibacterial activity by disrupting bacterial membrane potentials but also actively recruited osteoblasts and upregulated osteogenic differentiation pathways. This electromechanical stimulation essentially confers dual-functional properties to the barrier membrane without the requirement for loaded pharmaceutical drugs.^132^ To address the conflicting biological requirements of the tissue defect interface (e.g., inhibiting epithelial downgrowth while simultaneously promoting vascularization and osteogenesis), the field is advancing toward multiresponsive Janus (two-faced) membranes. These smart systems integrate structural compartmentalization with dynamic nanomaterials capable of responding to oxidative stress, immune imbalances, and pH variations. By simulating physiological signal gradients, these platforms are designed to provide self-regulating and self-limiting release mechanisms, which, if confirmed in clinical studies, could enhance the predictability of complex tissue reconstruction and reduce the frequency of clinical interventions.^133,134^

### 4.2 Fourth Dimension (4D) Bioprinting and Shape-Memory Materials

3D printing has revolutionized matrix preparation, but 4D printing represents the next evolutionary step. 4D printing uses smart “inks” to create structures that can change their shape, properties, or functions after fabrication in response to a stimulus.^135^ This technology enables the synthesis of devices with dynamic structural designs, capable of self-assembling or adapting over time. The main facilitator of this technology is shape-memory polymer (SMP), which can be programmed to hold a temporary shape and then return to their original and permanent shape under a specific stimulus, such as heat.^136^ The most immediate application of SMPs is the creation of membranes that can be implanted in a compact shape through minimally invasive surgery and, once in place, expand to perfectly cover a complex malformation. Furthermore, 4D printing can be used to create membranes whose internal structure changes over time.^137^ Jiang et al. explored exactly this, describing the development of self-adaptive implants whose porosity can gradually increase to allow cell permeation or whose stiffness can decrease as the new tissue becomes stronger.^95^ This programmed transformation allows the scaffold to dynamically adapt to the tissue’s needs in each phase of regeneration.^95^ The challenges for 4D printing are significant and include the need to expand the library of biocompatible, biodegradable shape-memory polymers and the complexity of accurately predicting shape transformations within the restricted, heterogeneous environment of the human body. A compelling future prospect is the printing of constructs that not only change shape but also undergo programmed maturation, with regions becoming bioactive or degrading at distinct times, thereby mimicking embryonic tissue development.^138^

### 4.3 Advanced Functionalization and Bioelectric Membranes

Functionalizing membranes with a single growth factor was a breakthrough, but tissue regeneration involves the complexity of multiple signals. Therefore, the research frontier lies in the creation of sequential and multifactorial release systems. Using multilayered membranes or different types of microparticles, it is possible to design the release of an anti-inflammatory agent in the first days, followed by an angiogenic factor and, finally, an osteogenic factor, thereby mimicking the natural healing cascade.^139,140^ Furthermore, creating concentration gradients of growth factors across the membrane can provide directional cues to guide cell migration and differentiation.^141^ Figure 3 outlines diverse strategies for surface functionalization and temporal release control. Methods such as physical adsorption, covalent binding, and polydopamine coating offer varying degrees of stability, enabling applications ranging from robust biomolecule immobilization to sustained release. Evolving from single-factor systems to multifactorial platforms, bioengineering now seeks to mimic physiological complexity through sequential or pulsatile release profiles, culminating in the development of bioelectric membranes that integrate physical and chemical stimuli. It is known that endogenous electric fields actively participate in cell signaling during healing and bone regeneration.^79^ To explore this phenomenon, piezoelectric materials such as PVDF, which generate electrical charges under mechanical stress, and conductive composites containing graphene, carbon nanotubes, or MXenes have been widely investigated. MXenes stand out as 2D nanomaterials with superior conductivity and promising biocompatibility.^142,143^ Preclinical assays demonstrate that local electrical stimulation promoted by these matrices favors the adhesion, proliferation, and differentiation of both osteoblasts and neural cells.^144^

**Figure 3:**
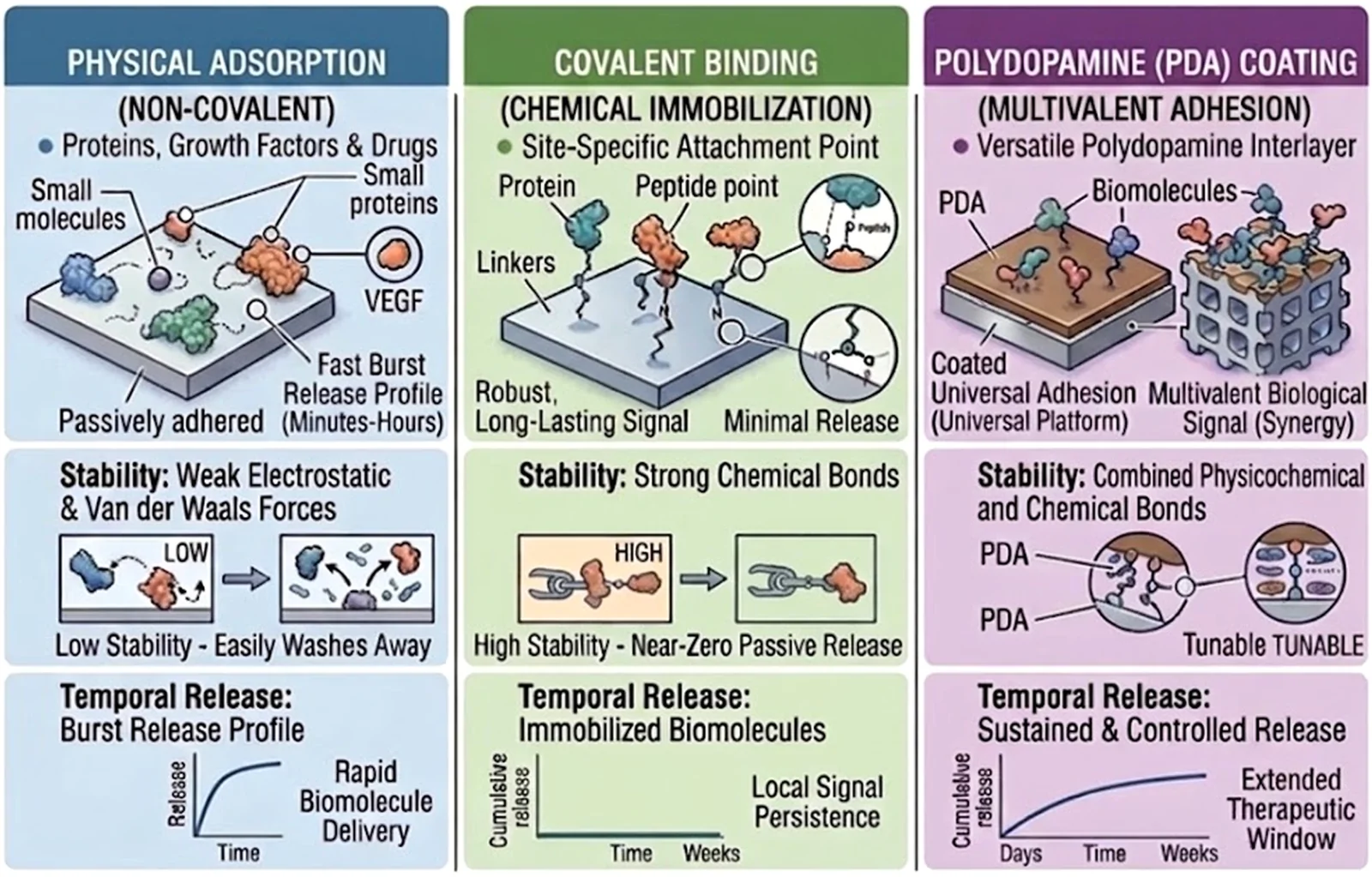
Diversity of strategies for surface functionalization and temporal release control. This image was created with Google AI Studio using the Nano Banana Pro model.

The debate surrounding bioelectric membranes focuses on the challenges of ensuring biocompatibility and long-term stability of the conductive components, as well as determining the ideal parameters for electrical stimulation for the target tissue. The future perspective involves integrating these systems with microchips and wireless energy technology, enabling the creation of “electroceutical” membranes capable of monitoring tissue state and applying stimuli on demand. Liao et al. highlighted that MXene-based conductive membranes emerge as a promising platform for neural regeneration due to their combination of high conductivity and biocompatibility.^145^

## 5 Limitations, Translational Challenges and Practical Insights

Despite significant advances in regenerative membrane technologies, the current evidence base remains largely limited to the preclinical stage, posing a major barrier to predicting clinical performance. Most studies rely on *in vitro* evaluations or small animal models that do not fully replicate the complexity of human tissue regeneration. *In vitro* studies frequently employ simplified media, such as phosphate-buffered saline, which maintains a stable pH but does not reproduce the enzymatic and cellular complexity of the inflammatory microenvironment. Although protein-enriched simulated fluids offer a better approximation, they still fail to mimic the metabolic dynamics of the injured tissues. Overcoming this gap requires validation in animal models that highlight such divergences. Additionally, standard *in vivo* animal models often fail to accurately replicate the enzymatic turnover rate and the inflammatory microbiome characteristic of human periodontal or bone defects, limiting the predictive value of the preclinical data. Even then, publication bias toward positive findings and the scarcity of standardized protocols for degradation and mechanical testing make quantitative comparisons between studies difficult, and most reported performances should be interpreted as best-case estimates. Therefore, caution is needed to extrapolate the preclinical results to clinical settings. These findings should be viewed as a proof of concept, with validation requiring well-conducted randomized clinical trials. Moreover, because the evidence base analyzed here combines in vitro studies, animal models, clinical trials, and reviews of different types, direct comparisons between membranes or between studies should be made with caution; for this reason, the level of evidence supporting each application is indicated explicitly throughout Section 3 and summarized in Table 3.

Despite the extensive *in vitro* development of novel polymeric membranes for guided tissue regeneration (GTR), the successful translation from bench to clinical market remains severely bottlenecked by industrial, regulatory, and patentability hurdles. Industrial scale-up of complex nanoarchitectures (e.g., electrospun nanocomposites or blended polymeric scaffolds) often compromises structural reproducibility during the transition to continuous, high-throughput manufacturing under good manufacturing practice (GMP) standards. Furthermore, terminal sterilization poses a critical paradox: conventional methods such as γ irradiation and ethylene oxide frequently induce polymer chain scission, thermal shrinkage, and premature degradation. These methods can severely degrade nanomechanical properties and alter the degradation kinetics of thermosensitive biopolymers.

The regulatory pathways of global agencies (e.g., FDA, European Medicines Agency) dictate the feasibility of commercializing new biomaterials. While passive barrier membranes are typically classified as Medical Devices, allowing for more predictable approval routes through agencies like the FDA, the current state-of-the-art focuses on bioactive membranes functionalized with active pharmaceutical ingredients, nanomicelles, or growth factors (e.g., rhBMP-2). This functionalization reclassifies the material as a combination product. Consequently, regulatory agencies mandate rigorous and highly expensive multicenter randomized clinical trials under stringent pathways (e.g., FDA Premarket Approval) to demonstrate both safety and the specific *in vivo* pharmacokinetics of the active compound. Similarly, the implementation of the new European Medical Device Regulation has practically abolished literature-based “clinical equivalence,” now requiring original, continuous postmarket clinical data even for standard devices.

Securing robust intellectual property for novel GTR formulations requires navigating increasingly stringent criteria to overcome the obviousness of the standard. Blending known polymers requires a clear demonstration of an unexpected synergistic technical effect. Furthermore, leading patent offices, including the European Patent Office and the United States Patent and Trademark Office, impose rigorous standards for biological patents, formulations, and second medical use. To obtain such patents, applicants must demonstrate a distinct mechanism of action supported by consistent experimental data within the claims. Rejections are systematic for patent applications that rely solely on broad theoretical claims or isolated *in vitro* cytotoxicity assays, so translational research must be comprehensively validated *in vivo* before intellectual property filing.

From a practical and functional standpoint, the membrane must have a mechanical profile that is specifically tailored to the functional demands of the implantation site. While bone regeneration requires materials with high stiffness and compressive strength to maintain space against soft tissue collapse, engineering dynamic tissues such as skin and blood vessels requires highly elastic polymers that can withstand cyclic deformations without structural failure. Currently, efforts aim to optimize these dynamic mechanical properties to avoid stress shielding, in which an excessively rigid implant absorbs the mechanical load, depriving bone tissue of the stimulus necessary for remodeling and leading to bone resorption. This concept is connected to the development of smart biomaterials and mechanical anisotropy. Electrospinning has become the primary strategy for manufacturing such structures because it enables the creation of nanofiber mats that mimic the physical architecture of the native extracellular matrix and thereby promote cell adhesion. The versatility of this method was demonstrated by Augustine et al. when they developed piezoelectric composite fibers containing zinc oxide that stimulated angiogenesis and by Patel et al. (2026), who proved that the precise alignment of nanofibers guides cell migration and increases tensile strength in tendon tissue engineering applications.^142,146^ The concept of material fatigue also deserves attention since membranes remain exposed to repeated load cycles during the regeneration period. Their strength cannot be evaluated solely under static loading; it must also account for the durability under prolonged mechanical stimuli. In this way, a more comprehensive view of the mechanical function of membranes is necessary, in which stiffness, flexibility, anisotropy, adaptive response, and fatigue resistance cannot be understood in isolation but rather as interdependent parameters that must be progressively adjusted throughout the regeneration process.

## 6 Conclusion and Future Perspectives

Membranes have evolved from passive barrier materials to increasingly sophisticated bioinstructive platforms that can actively modulate tissue regeneration. The present analysis demonstrates that successful membrane performance depends on a delicate balance among biocompatibility, mechanical integrity, biodegradation kinetics, and biological functionality. Importantly, no universal membrane exists, and optimal material selection remains strongly dependent on the target tissue and the clinical requirements.

Despite substantial progress, major challenges persist, including vascularizing large tissue defects, variability in biological responses, insufficient standardization across studies, and limited predictive value of many preclinical models. Therefore, statements about future therapeutic potential should be viewed as emerging research directions rather than established clinical evidence. Addressing these barriers, along with process scale-up and regulatory challenges, is crucial to accelerating the clinical translation. Future advances are expected to arise from personalized, intelligent membrane systems that integrate immunomodulatory design, sequential delivery of biological signals, conductive and bioelectric functionalities, and adaptive architectures enabled by 4D bioprinting. Such technologies may transform membranes into dynamic therapeutic platforms that can sense, interpret, and actively respond to tissue-specific regeneration demands. Consequently, future research should prioritize multifunctional systems combining biological efficacy, manufacturing scalability, regulatory compliance, and clinical applicability to fully realize the potential of regenerative medicine. Smart membranes, such as those incorporating colorimetric sensors, nanomaterials, and other responsive systems, represent promising conceptual advances for tissue engineering; however, because their efficacy is currently supported mainly by *in vitro* and animal models, their clinical translation will demand rigorous validation in future clinical trials to confirm their long-term safety and effectiveness in humans.

## 7 Methodology

This Review was structured as a narrative review of the literature to identify and critically evaluate the state of the art of membranes for tissue regeneration.

### 7.1 Literature Search Strategy

We searched PubMed/MEDLINE, Scopus, and Web of Science from January 2010 to May 2026 and supplemented the search by manually selecting seminal contributions published before 2010. The search strategy was designed for high sensitivity, combining two conceptual blocks with the Boolean operator (AND), one covering regenerative applications (e.g., tissue regeneration, tissue engineering, wound healing, osteogenesis, cartilage and nerve repair) and one covering membrane-related structures, materials, and technologies (e.g., membranes, scaffolds, films, dressings, chitosan, bacterial cellulose, collagen, PLGA, polycaprolactone, hydroxyapatite, titanium, electrospinning, 3D printing, stimuli-responsive, and piezoelectric systems), complemented by a third block (OR) of specific satellite topics relevant to the review (e.g., autologous chondrocyte implantation, pH-responsive systems, skin substitutes, cartilage defects, and antimicrobial peptides).

### 7.2 Study Selection

Given the broad, high-sensitivity search, study selection used a multistage, computer-assisted screening, where titles and abstracts were processed by bioinformatics and AI tools (Claude Fable 5), with human assessment reserved for full texts, as declared in this manuscript. After excluding duplicates, a first bioinformatic filter based on Boolean co-occurrence rules was applied to titles and abstracts, requiring the joint presence of a membrane/scaffold-related structural term and a regenerative application term, thereby excluding records from out-of-scope domains. The records were classified using AI-assisted semantic relevance on titles and abstracts, ranking them against the review scope to produce a short list. A final AI-assisted eligibility screening evaluated the shortlisted records against the inclusion and exclusion criteria, and records approved at this stage proceeded to human full-text assessment. Inclusion criteria comprised original research papers (*in vitro* and *in vivo*), narrative and systematic reviews, meta-analyses, and clinical trials published in English in peer-reviewed publications. Conference abstracts, nonpeer-reviewed papers, and studies not focused on membranes for tissue repair were excluded.

A limitation of the study selection is that AI-assisted screening was employed to reduce the initial volume of the retrieved records. Although the authors manually assessed all studies retained after this stage, they did not formally validate AI screening. Therefore, the possibility that some relevant studies were inadvertently excluded during the preliminary filtering process cannot be completely ruled out.

A total of 1,155,563 records were identified across the databases (PubMed, *n* = 306,803; Scopus, *n* = 410,361; and Web of Science, *n* = 438,399). After removal of duplicates (*n* = 524 042), 631,521 records underwent the bioinformatic co-occurrence filter, which retained 421,734 records. The AI-assisted semantic relevance classification reduced this set to 3275, of which 2863 were excluded by the AI-assisted eligibility screening for not meeting the eligibility criteria. The remaining 412 records were assessed by the authors for full-text eligibility, resulting in the exclusion of 279 records and inclusion of 133 records. These were complemented by six seminal studies published between 1982 and 2003, identified through citation tracking, and by six further papers cited directly to document the properties of specific materials, yielding a final corpus of 145 publications that formed the basis of the analysis presented in this review.

As a narrative review, a formal systematic assessment of the quality or risk of bias of the included studies was not performed. Consequently, studies with varying degrees of methodological rigor and different levels of evidence, ranging from early *in vitro* experiments and animal models to randomized clinical trials, were discussed without a standardized weighting. Because no single validated risk-of-bias instrument can be uniformly applied across *in vitro*, animal, clinical, and review-type studies, a qualitative hierarchy of evidence (clinical trials and systematic reviews > established clinical practice and case series > animal models > *in vitro* studies) was adopted instead, and the level of evidence supporting each membrane class is stated explicitly in Section 3 and summarized in Table 3. For this reason, statements regarding the clinical translation of the membrane technologies presented here should be interpreted with caution.

## Notes

Declaration of generative AI (artificial intelligence) and AI-assisted technologies in the writing process. In the course of preparing this work, the author(s) used ChatGPT (https://chat.openai.com/), Claude Fable 5, and Grammarly Inc. (grammarly.com) at some places to check grammar and improve readability. These tools were not used for generating information. After using an AI tool or service, the author(s) reviewed and edited the content as needed and assume full responsibility for the content of the publication. Google AI Studio (https://aistudio.google.com/), using the Nano Banana Pro model, was used to prepare the images shown in Figures 1, 2, and 3, as well as the Graphical Abstract.

## Abbreviations

AI: artificial intelligence
ACI: autologous chondrocyte implantation
DMF: dimethylformamide
d-PTFE: dense polytetrafluoroethylene
e-PTFE: expanded polytetrafluoroethylene
ECM: extracellular matrix
FDA: Food and Drug Administration
GMP: Good Manufacturing Practice
GBR: guided bone regeneration
GTR: guided tissue regeneration
GPR: guided periodontal regeneration
HA: hydroxyapatite
MACI: matrix-induced autologous chondrocyte implantation
NGCs: neural guidance conduits
PCL: polycaprolactone
PGA: poly­(glycolic acid)
PLA: poly­(l-lactic acid)
PLGA: (poly­(lactic-*co*-glycolic acid))
PTFE: polytetrafluoroethylene
PVA: poly­(vinyl alcohol)
PVDF: polyvinylidene fluoride
TCP: β-tricalcium phosphate
TGF-β1: transforming growth factor beta 1
VEGF: vascular endothelial growth factor
SMP_S_: shape-memory polymers
3D: three-dimensional and 4D, fourth dimension.
